# Cullen Sign Associated with External Iliac Artery Aneurysm Rupture: A Case Report

**DOI:** 10.5811/cpcem.24997

**Published:** 2025-01-12

**Authors:** Kendra M. Douglas, William R. Davis, Jaron D. Raper

**Affiliations:** University of Alabama at Birmingham, Department of Emergency Medicine, Birmingham, Alabama

**Keywords:** Cullen sign, aneurysm, iliac artery

## Abstract

**Introduction:**

Cullen sign is an area of periumbilical ecchymosis that results from blood tracking along the round ligament. Any source of retroperitoneal or abdominal hemorrhage can cause Cullen sign, but it is often described in association with acute pancreatitis.

**Case Report:**

Here we report a case of a chronically ill male who presented with a bulging sensation in his lower abdomen and lower abdominal pain. On physical examination this patient was noted to have a large area of periumbilical ecchymosis predominantly on the left aspect of the umbilicus, consistent with Cullen sign. Computed tomography abdomen and pelvis were remarkable for an enlarging left external iliac artery aneurysm with adjacent hematoma and multifocal intraperitoneal hematoma tracking into the right side of the abdomen, concerning for aneurysmal rupture. The patient was taken to the operating room for a left iliac artery arteriogram and stent placement.

**Conclusion:**

Isolated iliac artery aneurysms are rare and represent less than 2% of all abdominal aneurysmal disease; furthermore, external iliac artery aneurysms are exceedingly rare and account for the least common abdominal aneurysmal pathology. This case demonstrates the importance of considering other etiologies of Cullen sign beyond pancreatitis, including aneurysmal ruptures.

## INTRODUCTION

Cullen sign is an often taught but rarely observed complication of multiple intra-abdominal and retroperitoneal processes. First described in 1918 in association with a ruptured ectopic pregnancy, it is defined as subcutaneous edema and bruising around the umbilicus. Cullen sign has more recently been described in association with acute pancreatitis, where it is observed in 1–3% of patients.^1^,^2^ However, the etiologies causing Cullen sign include a variety of other sources of retroperitoneal and abdominal hemorrhage, among them rectal sheath hematomas, splenic rupture, and duodenal ulcer perforation.^3^

In this case we describe a patient who was noted to have Cullen sign caused by a large, ruptured, left external iliac artery pseudoaneurysm. While there are several case reports that attribute Cullen sign to aortic and internal iliac artery pathology, we are unaware of any report of Cullen sign related to a ruptured external iliac artery aneurysm.^4^

## CASE REPORT

A 75-year-old male with a past medical history of chronic obstructive pulmonary disease (COPD), peripheral artery disease, hypertension, hyperlipidemia, abdominal aortic aneurysm (previously repaired in 2015), and sarcoidosis presented to the emergency department for evaluation of lower abdominal pain and a bulging sensation in his lower abdomen. The patient reported he had been hospitalized two weeks prior to this presentation secondary to a COPD exacerbation, but he reported his dyspnea to be improving. Review of systems was otherwise negative.

**Image 1:**
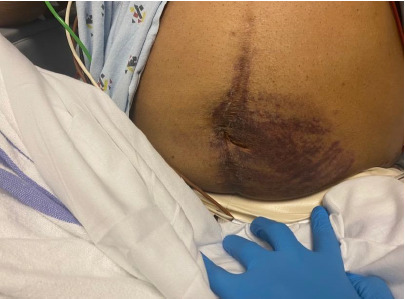
Periumbilical ecchymosis (Cullen sign).

**Image 2:**
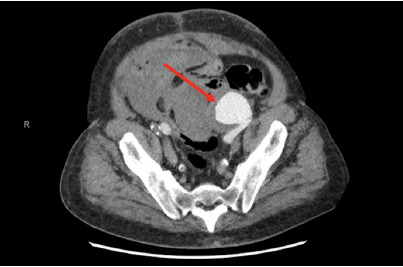
Left iliac artery aneurysm (arrow).

**Image 3:**
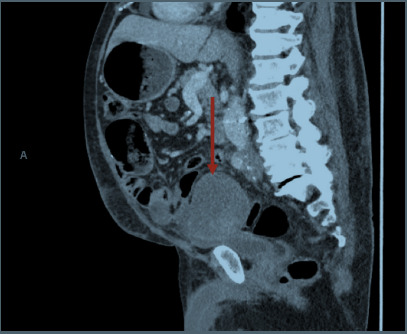
Intra-abdominal hematoma from aneurysmal rupture (arrow).

On the initial physical examination, the patient was in no acute distress and hemodynamically stable. His abdominal exam was remarkable for a large area of periumbilical ecchymosis predominantly on the left aspect of the umbilicus, consistent with Cullen sign (Image 1). The patient’s basic metabolic panel and liver enzymes were unremarkable. Hemoglobin was 10.9 grams per deciliter (g/dL), down from 14.2 g/dL (obtained two months prior) (reference range: 11.3 gm/dL–15.2 gm/dL). Computed tomography (CT) abdomen and pelvis with contrast demonstrated an enlarging left external iliac artery aneurysm with adjacent hematoma and multifocal intraperitoneal hematoma tracking into the right side of the abdomen, concerning for an interval aneurysmal rupture (Image 2 and Image 3). The patient’s hemoperitoneum and hematomas were entirely new since a previous positron emission tomography-CT obtained two months prior. Also reported was a stable 2.7-centimeter (cm) right common femoral artery pseudoaneurysm and patent infrarenal abdominal aorta iliac bypass grafts.

The patient was subsequently transferred to a regional tertiary care center for vascular surgery consultation. He was taken to the operating room for left iliac artery arteriogram with stent placement. Initially, the vascular surgery team exposed the left femoral artery and then performed an aortoiliac angiogram via the left iliac artery. Subsequently, vascular surgery placed a Viabahn 11 millimeter x 10 cm stent (WL Gore & Associates, Inc, Newark, DE) in the left external iliac artery. Left dorsalis pedis and posterior tibial pulses were confirmed via Doppler upon case completion.

## DISCUSSION

Cullen sign is an area of ecchymosis around the periumbilical region that results from blood tracking along the round ligament. This can occur with any hemorrhagic fluid from the retroperitoneal compartments when the fluid extends along the gastrohepatic and falciform ligaments to the umbilicus, allowing entry into the abdominal wall muscles. Usually, the abdominal wall musculature is isolated by various fascia and sheaths; however, when these protective structures are damaged or missing, blood may track into the subcutaneous region of the muscle.^3^

**CPC-EM Capsule**

What do we already know about this clinical entity?

*Cullen sign and iliac artery aneurysmal disease are well described clinical findings without known association*.

What makes this presentation of disease reportable?

*This case report demonstrates a relationship between Cullen sign and a ruptured iliac artery aneurysm, which has not been previously described*.

What is the major learning point?

*It is vital is to broaden the existing clinical differential for Cullen sign to include the emergent vascular pathology of iliac artery aneurysms*.

How might this improve emergency medicine practice?

*A heightened awareness of this association will help clinicians form their differential for patients presenting with Cullen sign*.

Isolated iliac artery aneurysms are rare, representing less than 2% of all abdominal aneurysmal disease; furthermore, external iliac artery aneurysms are exceedingly rare and account for the least common abdominal aneurysmal pathology.^5^ External iliac artery aneurysms can be isolated but can also be associated with more proximal aneurysms that have undergone extension. Traumatic or iatrogenic injury to the arterial wall can also lead to aneurysms but are also associated with pseudoaneurysm. The clinical presentation of an external iliac artery aneurysm can vary widely from being asymptomatic to symptoms of mass effect with venous occlusion and lower extremity edema to hemorrhage from rupture.^6^ In this case, the left external iliac artery aneurysm ruptured, causing intra-abdominal hemorrhage, and resulted in periumbilical ecchymosis (Cullen sign).

An additional physical exam characteristic often associated with pancreatitis is the Grey-Turner sign, which is seen as ecchymosis in the subcutaneous tissue of the flanks.^1^,^7^ Like Cullen sign it is a rare finding seen in severe intra-abdominal pathology including splenic rupture, retroperitoneal hemorrhage, or severe liver pathology. 8 The mechanism is like Cullen sign in which blood can traverse the typical intra-abdominal and abdominal wall fascia to become deposited within the subcutaneous tissue on the flank. The blood can take several days to accumulate and degrade to a point it can be appreciated on exam.^7^ Unfortunately, when disease processes have been able to develop long enough to manifest as either Grey-Turner or Cullen sign, they are often more severe.

The patient presented in this case was overall well appearing and did not have significant tenderness on abdominal exam. It was not until the skin on his abdomen was examined that the large areas of ecchymosis were revealed. Cullen sign was well demonstrated on exam, and it was the only exam characteristic that signaled how critically ill the patient could become. The rupture of his arterial aneurysm was relatively controlled at that time but could easily have worsened if proper treatment had been delayed. This case helps reiterate the importance of thorough physical exams and the role they can play in targeting or broadening a workup when a history or initial labs alone are reassuring.

While Cullen sign has been widely reported in the setting of pancreatitis, there are limited reports of other causes of this exam finding. When present, Cullen sign should be seen and interpreted as an indication of some type of either retroperitoneal or intra-abdominal hemorrhage, rather than simply a sign associated with pancreatitis, as previously thought. We are unaware of any other reports of Cullen sign associated with ruptured external iliac pseudoaneurysms.

## CONCLUSION

Cullen sign is an important clinical exam finding that indicates retroperitoneal or intraperitoneal hemorrhage, which can be caused by a variety of etiologies. We describe a previously unrecorded pathologic cause of Cullen sign. It is important for emergency physicians to maintain a broad differential when Cullen sign is present, as it may be indicative of any hemorrhagic source in the abdomen.
